# Monitoring the Site-Specific Solid-State NMR Data in Oligopeptides

**DOI:** 10.3390/ijms21082700

**Published:** 2020-04-13

**Authors:** Jiří Czernek, Jiří Brus

**Affiliations:** Institute of Macromolecular Chemistry, Czech Academy of Sciences, Heyrovsky Square #2, 16206 Prague, Czech Republic; brus@imc.cas.cz

**Keywords:** oligopeptides, solid-state NMR, plane-wave DFT, GIPAW, samarosporin

## Abstract

Reliable values of the solid-state NMR (SSNMR) parameters together with precise structural data specific for a given amino acid site in an oligopeptide are needed for the proper interpretation of measurements aiming at an understanding of oligopeptides’ function. The periodic density functional theory (DFT)-based computations of geometries and SSNMR chemical shielding tensors (CSTs) of solids are shown to be accurate enough to support the SSNMR investigations of suitably chosen models of oriented samples of oligopeptides. This finding is based on a thorough comparison between the DFT and experimental data for a set of tripeptides with both ^13^C_α_ and ^15^N_amid_ CSTs available from the single-crystal SSNMR measurements and covering the three most common secondary structural elements of polypeptides. Thus, the ground is laid for a quantitative description of local spectral parameters of crystalline oligopeptides, as demonstrated for the backbone ^15^N_amid_ nuclei of samarosporin I, which is a pentadecapeptide (composed of five classical and ten nonproteinogenic amino acids) featuring a strong antimicrobial activity.

## 1. Introduction

Oligopeptides have been intensely studied due to their numerous applications [1]. One of the most important areas of the research aims at finding an alternative antimicrobial strategy [2]. Related host-defense oligopeptides [3], mostly of the peptaibol family [4], have been analyzed in terms of their function, which is based on an ability to form pores in biological membranes [5]. The solid-state nuclear magnetic resonance (SSNMR) measurements are crucial in understanding the pore formation properties of peptaibols [6]. Due to the large size and complexity of peptaibols and similar oligopeptides, their SSNMR investigations usually do not use the site-specific spectroscopical/structural data for every investigated peptide unit. Instead, either of the two strategies is adopted. In the first approach, “collective” values for a group of nuclei are employed (for example, for the ^15^N chemical shift tensors (CSTs) of amidic nitrogens in classical amino acid and α-amino isobutyric acid (Aib) residues, thus neglecting differences of CSTs within each group [7]). In the second approach, site-specific results are obtained only for some atoms, which are isotope-labeled (see impressive efforts by the group of Naito to study eleven alamethicin molecules singly labeled with ^13^C at the position of respective carbonyl carbon [8]), or chemically introduced in case of ^19^F [9]. Importantly, the plane wave density functional theory (PW DFT)-based calculations of periodic structures and their NMR properties [10] can currently be applied even to bigger oligopeptides of up to about 20 amino acids (depending on the number of formula units in the crystal’s unit cell), and may provide useful information about differences in the local geometry and SSNMR parameters within, for instance, the whole backbone of a peptaibol [11]. It is thus of interest to calibrate the results of the PW DFT computations against highly accurate measurements, such as those described below, which were taken from single crystal studies [12,13,14], in order to establish if it could be possible to reliably predict a site-specific variation in the SSNMR data and include this information in analyses of experiments on larger oligopeptides [15]. This type of monitoring is presented here for samarosporin I (a naturally occurring peptaibol comprised of 15 amino acids [16]) on the basis of benchmarking calculations for a set of six triglycines, and for *N*-Ac-Aib-OH, *N*-Ac-Leu-OH and Ala-Pro-Gly dihydrate, after their assessment performed for melanostatin (Pro-Leu-Gly-NH_2_ hemihydrate [17]). The results directly capture an influence of secondary structural elements upon the NMR parameters (see reference [18] for the most recent review of this topic) and could be important in NMR crystallography [19,20,21,22,23] of oligopeptides and in an interpretation of spectra of their oriented samples [24]. 

## 2. Results

### 2.1. The Chemical Shielding Tensors of Triglycines

**Figure 1:**
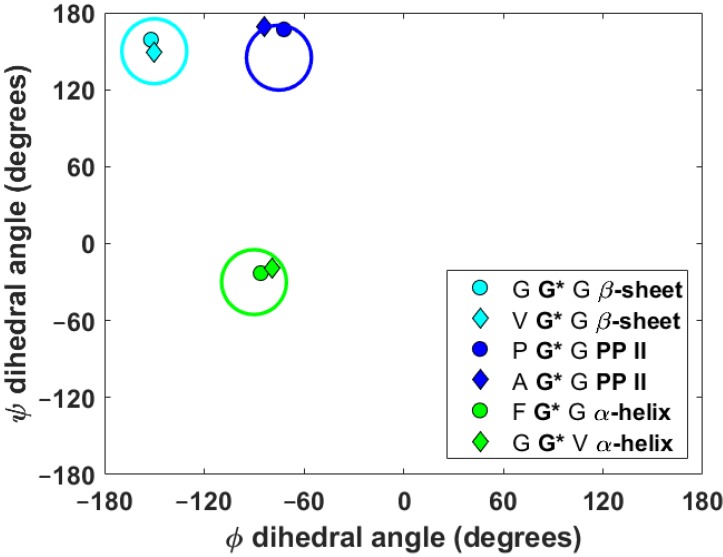
The Ramachandran plot for central glycyl residues in the set of tripeptides. Typical β-sheet, polyproline II (PP II) and α_R_-helical regions are schematically shown by large cyan, blue and green circles centered at [−150°; +150°], [−75°; +145°] and [90°; −30°], respectively.

Some time ago, Wittebort et al. performed meticulous single-crystal (SC) SSNMR measurements of central glycyl ^13^C_α_ and ^15^N_amid_ CSTs in two relatively large series of tripeptides of known solid-phase geometry, featuring the torsion angles typical for common secondary structural motifs found in polypeptides [12,13,14]. Here, six tripeptides (specified in Table 1 using their simplified names in apostrophes) are taken from the sets studied by Wittebort et al. so that both the ^13^C_α_ and ^15^N_amid_ CSTs are available for two representatives of each of, arguably, the most frequent regular secondary structures: β-sheet, α_R_-helix, and the polyproline II (PP II) helix [25]. Figure 1 presents the Ramachandran plot of the {φ, ψ} angles obtained from the PW DFT optimization (see Part 4) of these six tripeptides.

**Figure 2:**
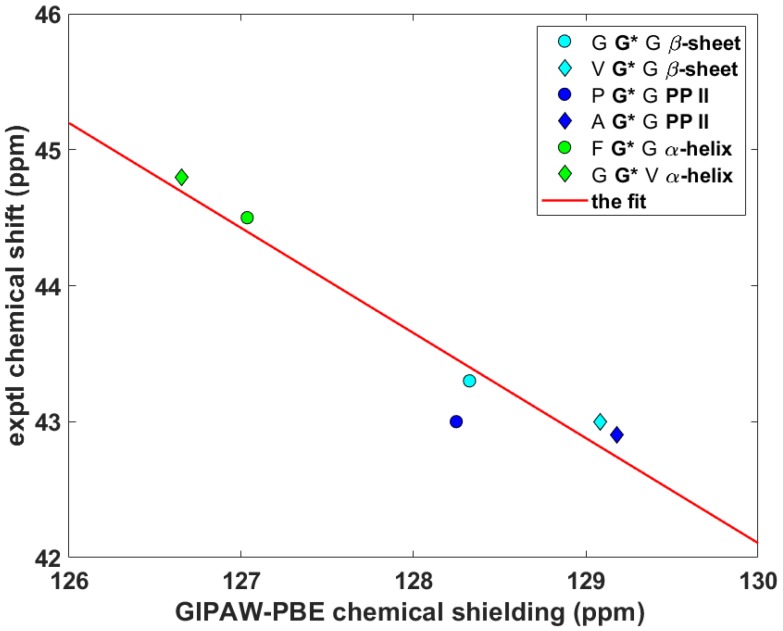
The isotropic ^13^C data of C_α_ nuclei of selected glycyls in tripeptides.

**Figure 3:**
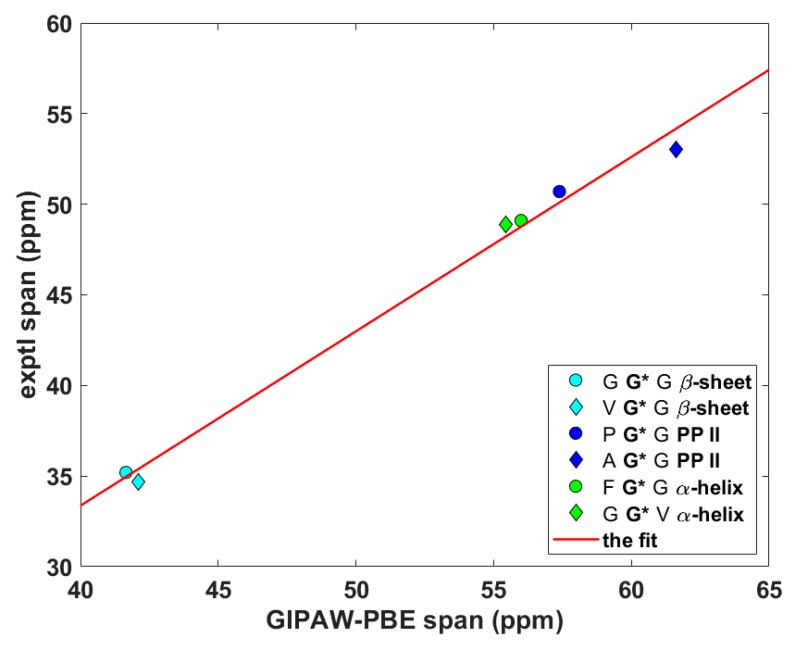
The anisotropic ^13^C data of C_α_ nuclei of selected glycyls in tripeptides.

**Figure 4:**
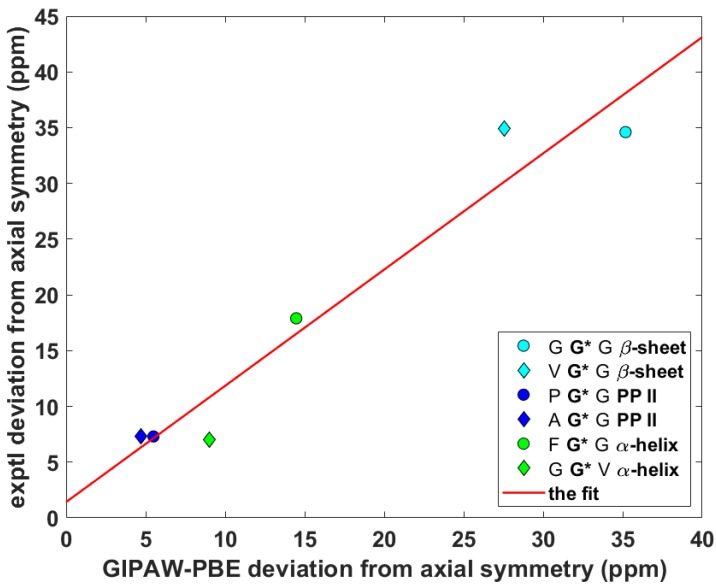
The anisotropic ^15^N data of N_amid_ nuclei of selected glycyls in tripeptides.

Wittebort et al. [12,13] discerned three important trends in the dependence of SC SSNMR parameters upon the secondary structure. First, they found that the ^13^C_α_ isotropic chemical shifts, δ^iso^, of glycyls were always lower in α-helices than in β-sheets and PP II helices. Second, values of the span, δ^span^, of ^13^C_α_ CSTs, δ^span^ = δ_33_ – δ_11_ for δ_11_ ≤ δ_22_ ≤ δ_33_ ordering of the eigenvalues of the chemical shift tensor, were the highest for glycyls in PP II, followed by those in α-helices and then by those in β-sheets. Third, values of the deviation from the axial symmetry, δ^dev^, of ^15^N_amid_ CSTs, δ^dev^ = δ_22_ – δ_11_ for the same ordering as above, were the highest for glycyls in β-sheets, followed by those in PP II and then by those in α-helices. Thus, an unambiguous identification of the secondary structure of a peptide should be possible by combining the three experimental trends [13]. Here, the ability of the gauge-including projector augmented wave (GIPAW) Perdew–Burke–Ernzerhof (PBE) calculations (see Materials and Methods section) of the chemical shielding tensors to reproduce the aforementioned tendencies is tested. The eigenvalues, both theoretical and experimental, obtained for the six tripeptides are summarized in Supporting Materials Tables S1 (^15^N_amid_) and S2 (^13^C_α_). The ^13^C_α_ δ^iso^ and δ^span^, and ^15^N_amid_ δ^dev^ SSNMR parameters are graphically shown in Figure 2, Figure 3 and Figure 4 (fits of the eigenvalues are included in Supporting Materials). Expectedly [26], these figures illustrate a full agreement between the measured dependences and their theoretical counterparts, which were obtained from the GIPAW PBE calculations of the chemical shielding after the PW PBE optimizations of crystalline peptides’ geometries.

### 2.2. The Calibration of the ^15^N Chemical Shielding for Peptides

**Figure 5:**
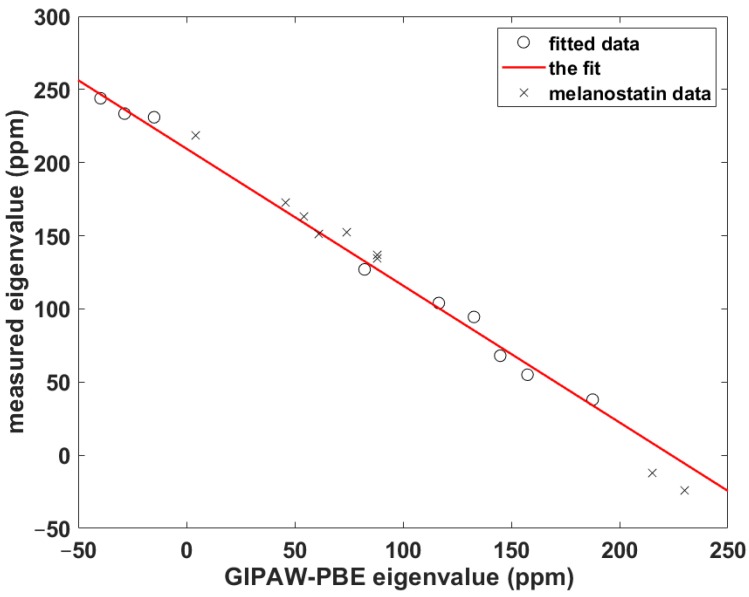
The linear relationship between the ^15^N_amid_ *δ_ii_* and *σ_ii_* data described in the text.

For cases when the experimental information is incomplete or currently unavailable, it is crucial to be able to quantitatively predict the parameters of the ^15^N_amid_ chemical shift tensor, namely, its eigenvalues and their orientation in the molecular frame, for their further use in simulations of the SSNMR spectra of peptaibols (see the review [27] and references cited therein). Importantly, an unbiased calibration of the relationship between the chemical shift and computed chemical shielding data needs to include some non-canonical amino acid(s), because peptaibols contain quaternary residues such as l-4-hydroxyproline (Hyp), d-isovaline (Iva) or the aforementioned Aib, while experimental studies of peptides containing those residues are scarce [7]. As a consequence, the benchmark set consists of just three systems: *N*-Ac-Aib-OH and *N*-Ac-Leu-OH from the SSNMR study of powders [7] and the prolyl data of Ala-Pro-Gly dihydrate obtained by Wittebort et al. [14]. The ability to accurately predict the ^15^N_amid_ chemical shift tensor components using this benchmark set is verified for Pro, Leu and Gly sites of melanostatin (it is noted that in reference [17] the experimental data were reported in nitromethane scale [28] and in the icosahedral representation [29]). The computational procedure is based on our previous work [30], and it should be realized that it does not explicitly use a chemical shielding of any referencing species. It involves fitting of a set of *δ_ii_* to the corresponding set of *σ_ii_* to obtain the slope, *a*, and the intercept, *b*, in {*σ_ii_*} = a*{*δ_ii_*} + *b*, as illustrated in Figure 5 (in this shorthand notation, curly brackets indicate correctly ordered elements of both sets; see also reference [31]). Subsequently, the theoretical chemical shift, *ε*^iso^, of a given nucleus is estimated from *ε*^iso^ = (*ε*_11_ + *ε*_22_ + *ε*_33_)/3, where *ε_ii_* = a*σ_ii_ + *b*. In the present case, *a* = –0.93574, *b* = 209.54 ppm, adjusted *R*^2^ = 0.99359, one standard deviation = 6.1 ppm (the underlying data are gathered in Table S1). As follows from an inspection of Table 2, the differences are minimal (smaller than one ppm) between the measured and theoretical isotropic chemical shifts of the amidic nitrogens in melanostatin. Hence, this calibration is used to estimate the ^15^N_amid_ chemical shift data of samarosporin I that are discussed below.

Regarding an orientation of the CSTs in the molecular/crystal frame, we recently showed the GIPAW PBE calculations to provide highly accurate results for N_δ_ and N_ε_ sites in l-histidine hydrochloride monohydrate [32]. Nevertheless, the SC SSNMR measurements by Wittebort et al. [14] of the central glycyl ^15^N_amid_ sites in AGG and GGV and of the prolyl ^15^N_amid_ in APG (see Table 1) are employed here to confirm the quality of the PW DFT predictions. The experimental values of the set of angles from reference [14] are shown in Table 3 together with the corresponding theoretical results (reconstructed using the specific peptide plane definitions given below Table 2 of reference [14]), which can be seen to be quantitatively correct.

### 2.3. Predictions for Samarosporin I

Samarosporin I is the subfamily 2 peptaibol [33]. Its sequence is Ac-Phe-Aib-Aib-Aib-Val-Gly-Leu-Aib-Aib-Hyp-Gln-Iva-Hyp-Aib-Fol (Aib, Hyp, and Iva were defined above, while Ac and Fol, respectively, denote acetyl and phenylalaninol at the *N*-end and *C*-end of the peptide). Samarosporin I, due to its strong antimicrobial activity [34], was studied by SC X-ray diffraction (XRD) at two temperatures (100 and 293 K) in order to help elucidate its mechanism of action [35]. An SSNMR investigation of this oligopeptide is clearly desirable and would benefit from the monitoring of the CSTs if, for instance, the so-called polarity index slant angle (PISA) wheels [36] were to be analyzed (see reference [37] for a survey of related experimental techniques). Specifically, the solid-phase structure of samarosporin I features right-handed helical folding. The helix tilt angle could thus be determined in oriented lipid bilayers from two-dimensional spectra correlating ^1^H_amid_–^15^N_amid_ dipolar couplings and ^15^N_amid_ chemical shift anisotropy of atoms in peptide planes in a α-helical configuration (for details, see reference [37]). The relevant structural information about the samarosporin I backbone is collected in Table 4, namely, the values obtained after the PW PBE optimization of the N_amid_–H_amid_ bond lengths, r_NH_, and the {φ, ψ} dihedral angles. The {φ, ψ} values reported in Table 3 of reference [16] are also shown in Table 4 and indicate a close agreement between the DFT optimized and SC XRD structures of this oligopeptide. Additional structural information can be gleaned directly from the coordinates included as a PDB file in the Supporting Materials. The ^15^N_amid_ CSTs are characterized in terms of the following parameters: an estimate, *ε*^iso^, of the isotropic chemical shift; estimates, {*ε*_11_, *ε*_22_, *ε*_33_}, of principal components of the chemical shift tensor; the angles, {α, β, γ}, which describe an orientation of the CST in the crystal frame and are defined in Part 4 (numbering of atoms belonging to the respective reference planes is provided in Table S3). These results are further discussed in the next section.

## 3. Discussion

**Figure 6:**
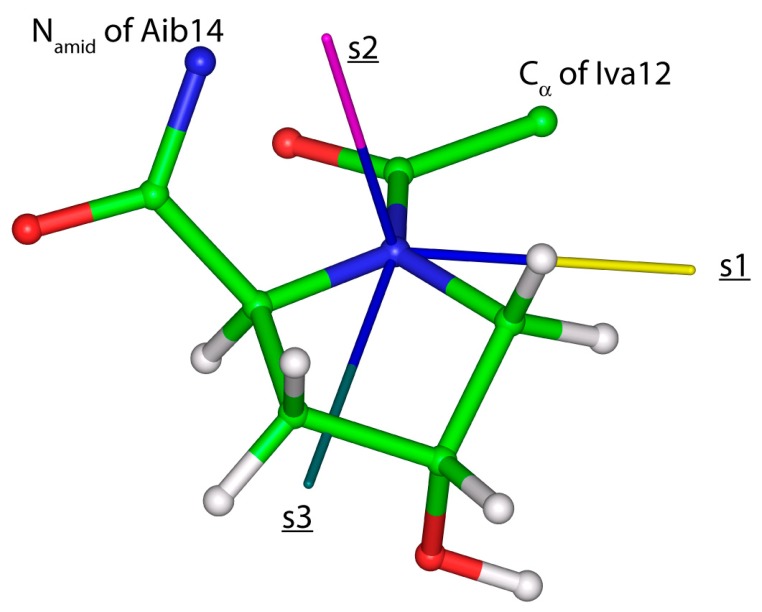
The fragment of samarosporin I with visualized eigenvectors associated with the eigenvalues of the ^15^N_amid_ chemical shielding tensor of Hyp13 residue (an eigenvector pertaining to the smallest, mid- and highest eigenvalue is colored in yellow, pink and dark green, respectively, and accordingly denoted as s1, s2 and s3).

High-accuracy data are needed for establishment of a quantitatively accurate relationship between the structure of oligopeptides and their SSNMR parameters. Here, the data are carefully chosen from the results of the {^13^C, ^15^N} SC measurements previously performed for the central (^13^C_α_ and ^15^N_amid_) glycyl nuclei in tripeptides with known crystal geometries belonging to any of the three most common secondary structural elements found in proteins. This set of structures is employed to investigate the predictive power of the PW DFT computations, which is found to be strong for the key SSNMR parameters (^13^C_α_ δ^iso^ and δ^span^, and ^15^N_amid_ δ^dev^). In addition, orientations of the ^15^N_amid_ CSTs are verified to be reliably described by the GIPAW PBE calculations carried out for the optimized geometries of AGG, GGV, and PGG. Hence, the predictive power of the PW DFT is used to calibrate the dependence of the chemical shift upon the computed chemical shielding (detailed in Section 2.2) and subsequently monitor the eigenvalues and orientations of the ^15^N_amid_ CSTs along the backbone of an antimicrobial peptide samarosporin I. Importantly, the computations showed the same trend as was discerned experimentally for other peptaibols [38], namely, the ^15^N_amid_ isotropic chemical shifts in Aib are significantly (by ca. 15 ppm in the present case) higher than in classical residues. An inspection of the averaged {*ε*_11_, *ε*_22_, *ε*_33_} values for these two groups of amino acids reveals the differences of the respective principal components leading to the distinctive isotropic chemical shifts. Specifically, the (“Aib”–“canonical”) differences, rounded to one ppm accordingly, amount to 14, 12, and 20 ppm for the *ε*_11_, *ε*_22_, and *ε*_33_ principal components. As for an orientation in the crystal frame of the ^15^N_amid_ CSTs, given by a {α, β, γ} triple of angles, it should be mentioned that the most important are the values of β. This is because they directly enter a simulation of the PISA wheels [35] (and the values of α are in a typical case assumed to be zero). In the present case, both β and α angle values are, in general, similar to those found in a computational study of another oligopeptide, ampullosporin A [11]. Namely, β angles are slightly higher in proteinogenic amino acids (median of 20.1°) than in Aib (median of 15.3°) sites of samarosporin I, and α angles are negligibly small, with values below 5°. It is also worth mentioning that the estimated ^15^N_amid_ chemical shift tensor components in hydroxyproline residues (Hyp10 and Hyp13) are close to the values found for nonproteinogenic amino acids (see Table 4), and hydroxyproline sites feature a specific orientation of their ^15^N_amid_ CST. In particular, the eigenvector associated with the least shielded eigenvalue is significantly (by more than 20°) tilted off the peptide plane (see Figure 6). This information could be useful in future studies of prolyl-containing peptides [39].

## 4. Materials and Methods

A computational approach was adopted that applies Kohn–Sham DFT in the pseudopotential PW scheme and imposes periodic boundary conditions to treat the investigated crystal structure as an infinite system, as detailed in references [40,41,42]. First, the starting geometries specified in Table 1 were subjected to the optimization of all atoms’ positions with respect to the crystal lattice energy approximated with the Perdew–Burke–Ernzerhof (PBE) [43] exchange-correlation functional, while the unit-cell parameters were fixed at their corresponding XRD values. Subsequently, the CSTs were predicted employing the gauge-including projector augmented wave (GIPAW) [44,45] method combined with the PBE functional. The CASTEP 6.1 suite of codes [42] was used with the pseudopotentials generated on-the-fly, and with the thresholds and settings consistent with the “fine” level of accuracy of Materials Studio 5.0 software (the technical assistance was provided by Dr. M. Hušák, University of Chemistry and Technology, Prague, The Czech Republic. In particular, the cut-off energy of the plane-waves was set to 550 eV in all the above-mentioned calculations.

The optimized crystal geometries and the eigenvectors associated with respective eigenvalues were adopted to establish an orientation of the investigated ^15^N_amid_ CSTs in the frame of the peptide bond. The reference plane was defined by the positions of N_amid_, H_amid_, and C_α_ atoms of the given amino acid and using N_amid_–H_amid_ and N_amid_–C_α_ bond vectors. Then, the angle α is defined by a projection onto this plane of the eigenvector ξ_1_ associated with the most shielded eigenvalue, σ_11_; the angle β is subtended between the ξ_1_ and the related N_amid_–H_amid_ bond vector; and γ is the angle between a normal to the N_amid_; H_amid_; C_α_ plane and the eigenvector ξ_2_ associated with the mid-shielded eigenvalue, σ_22_, of the ^15^N_amid_ chemical shielding tensor in question. The eigenvectors were processed by the INFOR software [46] for their visualization in a crystal/molecular frame.

## Figures and Tables

**Table 1:** Peptides investigated in this work.

Compound	Initial Geometry	SSNMR Data
Gly-Gly-Gly*HCl ‘GGG’	1110119 ^1^	from refs [12,13]
Val-Gly-Gly ‘VGG’	1129567 ^1^	from refs [12,13]
Pro-Gly-Gly ‘PGG’	1151185 ^1^	from refs [12,13]
Ala-Gly-Gly*H_2_O ‘AGG’	1119938 ^1^	from refs [12,13]
Phe-Gly-Gly ‘FGG’	1157783 ^1^	from refs [12,13]
Gly-Gly-Val*2H_2_O ‘GGV’	1134084 ^1^	from refs [12,13]
*N*-Ac-Aib-OH	1130667 ^1^	from ref. [7]
*N*-Ac-Leu-OH	624793 ^1^	from ref. [7]
Ala-Pro-Gly*H_2_O	1160528 ^1^	from ref. [14]
melanostatin (see text)	216376 ^1^	from ref. [17]
samarosporin I (see text)	4G14 ^2^	predicted

^1^ The Cambridge Crystallographic Database identifier (https://www.ccdc.cam.ac.uk/). ^2^ The Protein Data Bank identifier (https://www.rcsb.org/).

**Table 2:** The chemical shift/shielding tensor data (in ppm) of the ^15^N_amid_ nuclei in melanostatin. The σ values are taken from the CASTEP output, ε values are obtained from the parametrization as described in the text, and δ values are from the experiment [17].

Tensor Component		Site		
		Pro	Leu	Gly
the most shielded	σ	−11.8552	−23.9238	134.6846
	ε	220.6	231.9	83.5
	δ	215.1	229.9	88.2
the mid-shielded	σ	152.3491	136.9511	172.5634
	ε	67.0	81.4	48.1
	δ	74.1	88.1	45.9
the least shielded	σ	163.1567	151.2735	218.4587
	ε	56.9	68.0	5.1
	δ	54.4	61.2	4.1
isotropic part	σ^iso^	101.2169	88.1003	175.2326
	ε^iso^	114.8	127.1	45.6
	δ^iso^	114.5	126.4	46.1

**Table 3:** The comparison of calculated and single-crystal (SC) solid-state NMR (SSNMR) (taken from reference [14] and shown in parentheses) angles describing an orientation of the ^15^N_amid_ chemical shielding/shift tensor of the central residue (marked with an asterisk) in the peptide’s crystal frame. Designation of angles is the same as in reference [14] and should not be confused with the angles used in this work.

Site	Angle (in Degrees) ^1^		
	γ	β	φ
A G* G	1.5 (1)	18.8 (23)	34.8 (36)
G G* V	7.4 (11)	23.4 (20)	15.8 (15)
A P* G	4.0 (5)	23.3 (23)	4.2 (5)

^1^ ±3˚measurement uncertainty was reported in reference [14] for all the angles.

**Table 4:** Selected parameters of the samarosporin I backbone (values in parentheses are from the XRD study [16]). The N_amid_–H_amid_ distances (r_NH_) are in picometers, all angles in degrees, and {*ε*_11_, *ε*_22_, *ε*_33_, *ε*^iso^} data in ppm.

Site	r_NH_	φ	ψ	ε^iso^	ε_11_	ε_22_	ε_33_	α	β	γ
Phe1	101.73	−128 (−126)	−12 (−6)	115.5	44.3	79.8	222.3	0.4	17.8	5.9
Aib2	102.83	−51 (−52)	−43 (−47)	135.7	74.4	81.5	251.2	1.2	13.4	49.4
Aib3	103.97	−53 (−55)	−39 (−38)	125.8	72.7ε	76.2	228.6	1.3	18.0	35.2
Aib4	102.50	−55 (−55)	−47 (−50)	121.5	58.9	81.0	224.5	4.2	16.3	33.7
Val5	102.35	−76 (−78)	−45 (−41)	110.2	49.4	71.6	209.5	4.2	20.4	28.8
Gly6	102.64	−61 (−62)	−35 (−39)	108.8	45.0	61.1	220.4	1.0	20.1	48.4
Leu7	102.98	−75 (−73)	−38 (−36)	114.0	46.7	67.7	227.7	3.6	18.0	15.7
Aib8	102.08	−68 (−66)	−38 (−39)	122.6	56.7	79.2	231.9	1.0	11.7	29.6
Aib9	102.76	−52 (−54)	−47 (−44)	124.7	69.7	83.3	221.0	1.6	19.5	39.7
Hyp10	–	−65 (−64)	−13 (−16)	127.6	43.7	119.8	219.2	^1^	^1^	^1^
Gln11	103.01	−87 (−87)	−11 (−11)	107.2	48.7	59.3	213.6	2.6	20.6	44.5
Iva12	102.72	−54 (−53)	−41 (−40)	123.8	63.5	77.6	230.4	1.1	17.2	19.4
Hyp13	–	−67 (−67)	−10 (−11)	127.5	50.2	113.4	218.8	^1^	^1^	^1^
Aib14	102.94	−50 (−53)	−28 (−25)	128.1	66.7	80.2	237.3	1.1	14.3	49.7
Fol15	102.87	−66 (−65)	–	118.3	50.3	80.9	223.6	1.8	20.9	44.7

^1^ The definition of {α, β, γ} angles does not apply to hydroxyproline residues.

## Supplementary Materials

Supplementary materials can be found at https://www.mdpi.com/1422-0067/21/8/2700/s1.
